# Comparison of Deep Learning Approaches Using Chest Radiographs for Predicting Clinical Deterioration: Retrospective Observational Study

**DOI:** 10.2196/67144

**Published:** 2025-04-10

**Authors:** Mahmudur Rahman, Jifan Gao, Kyle A Carey, Dana P Edelson, Askar Afshar, John W Garrett, Guanhua Chen, Majid Afshar, Matthew M Churpek

**Affiliations:** 1 Department of Medicine, University of Wisconsin-Madison, 610 Walnut St, Madison, WI, 53792, United States, 1 608-262-9564; 2 Department of Biostatistics and Medical Informatics, University of Wisconsin-Madison, Madison, WI, United States; 3 Department of Medicine, University of Chicago, Chicago, IL, United States; 4 Department of Radiology, University of Wisconsin-Madison, Madison, WI, United States

**Keywords:** chest X-ray, critical care, deep learning, chest radiographs, radiographs, clinical deterioration, prediction, predictive, deterioration, retrospective, data, dataset, artificial intelligence, AI, chest, patient, hospitalized

## Abstract

**Background:**

The early detection of clinical deterioration and timely intervention for hospitalized patients can improve patient outcomes. The currently existing early warning systems rely on variables from structured data, such as vital signs and laboratory values, and do not incorporate other potentially predictive data modalities. Because respiratory failure is a common cause of deterioration, chest radiographs are often acquired in patients with clinical deterioration, which may be informative for predicting their risk of intensive care unit (ICU) transfer.

**Objective:**

This study aimed to compare and validate different computer vision models and data augmentation approaches with chest radiographs for predicting clinical deterioration.

**Methods:**

This retrospective observational study included adult patients hospitalized at the University of Wisconsin Health System between 2009 and 2020 with an elevated electronic cardiac arrest risk triage (eCART) score, a validated clinical deterioration early warning score, on the medical-surgical wards. Patients with a chest radiograph obtained within 48 hours prior to the elevated score were included in this study. Five computer vision model architectures (VGG16, DenseNet121, Vision Transformer, ResNet50, and Inception V3) and four data augmentation methods (histogram normalization, random flip, random Gaussian noise, and random rotate) were compared using the area under the receiver operating characteristic curve (AUROC) and the area under the precision-recall curve (AUPRC) for predicting clinical deterioration (ie, ICU transfer or ward death in the following 24 hours).

**Results:**

The study included 21,817 patient admissions, of which 1655 (7.6%) experienced clinical deterioration. The DenseNet121 model pretrained on chest radiograph datasets with histogram normalization and random Gaussian noise augmentation had the highest discrimination (AUROC 0.734 and AUPRC 0.414), while the vision transformer having 24 transformer blocks with random rotate augmentation had the lowest discrimination (AUROC 0.598).

**Conclusions:**

The study shows the potential of chest radiographs in deep learning models for predicting clinical deterioration. The DenseNet121 architecture pretrained with chest radiographs performed better than other architectures in most experiments, and the addition of histogram normalization with random Gaussian noise data augmentation may enhance the performance of DenseNet121 and pretrained VGG16 architectures.

## Introduction

Clinical deterioration is common in hospitalized patients and can lead to adverse outcomes, including increased morbidity and mortality if not identified and managed properly [1]. The early detection of patient deterioration and timely intervention can improve patient outcomes [2]. Various early warning scores (EWS) have been developed to identify the deterioration risk by monitoring different clinical variables, and the implementation of machine-learning EWS, such as the electronic cardiac arrest risk triage (eCART) score, has been associated with improved mortality [3-6]. Current EWS rely on structured data, such as vital signs and laboratory values, to predict clinical deterioration and ignore other data modalities that could potentially enhance prediction accuracy [7]. This results in lower detection and higher false-positive rates for these scores that could be mitigated by incorporating additional modalities [8].

Because respiratory failure is a common cause of clinical deterioration, the use of computer vision models with chest radiographs is a promising direction for improving EWS performance [9]. Although traditional computer vision models have historically been used to analyze chest radiographs, prior work on chest radiographs is limited to identifying specific diagnoses [10-12]. In some recent studies, chest radiographs are used to detect lung disease [[13,14]], acute respiratory distress syndrome [15], pneumonia [16,17], tuberculosis [18,19], and COVID-19 [20]. However, to facilitate other tasks with comprehensive machine understanding, chest X-ray interpretation models are being more commonly used with the help of computer vision and transformer-based natural language processing models [21,22]. The advancements in predictive analytics with deep learning methods have led to increased capabilities to extract meaningful information from medical images, including chest radiographs [23]. However, deep learning models have never been trained with chest radiographs to predict clinical deterioration outside the intensive care unit (ICU). There are numerous deep learning architectures for chest radiograph prediction models, such as VGG16, ResNet50, DenseNet121, and Vision Transformer, and the performance of these models is unknown for this specific task. Additionally, there are different data augmentation techniques available to further enhance the performance of a vision model by improving model generalization, but it is unknown whether these data augmentation techniques would improve the performance of the prediction model for this task.

To address these knowledge gaps, the objective of this study was to compare different computer vision architectures and augmentation methods with chest radiographs for predicting clinical deterioration. Our training pipeline incorporates extensive hyperparameter tuning through Bayesian optimization and validates the generalizability of models in a separate hold-out test set. The findings of our experiments have important implications for researchers developing computer vision deep learning models for clinical applications with chest radiographs.

## Methods

### Ethical Considerations

The study protocol was reviewed and approved by the University of Wisconsin Institutional Review Board (approval #2019‐1258). This study was a secondary analysis of limited HIPAA data from hospital electronic health records. The study was approved with a waiver of informed consent.

All direct identifiers of patients whose data were used in this study were de-identified prior to analysis to ensure participants privacy and confidentiality. Minimal necessary identifiable information was accessed or stored during the study beyond possible HIPAA data in clinical notes, radiological images, and real dates.

Participants did not receive any compensation for this data analysis, as no new data were collected and no direct contact with participants occurred.

### Study Population and Data Collection

**Figure 1.**
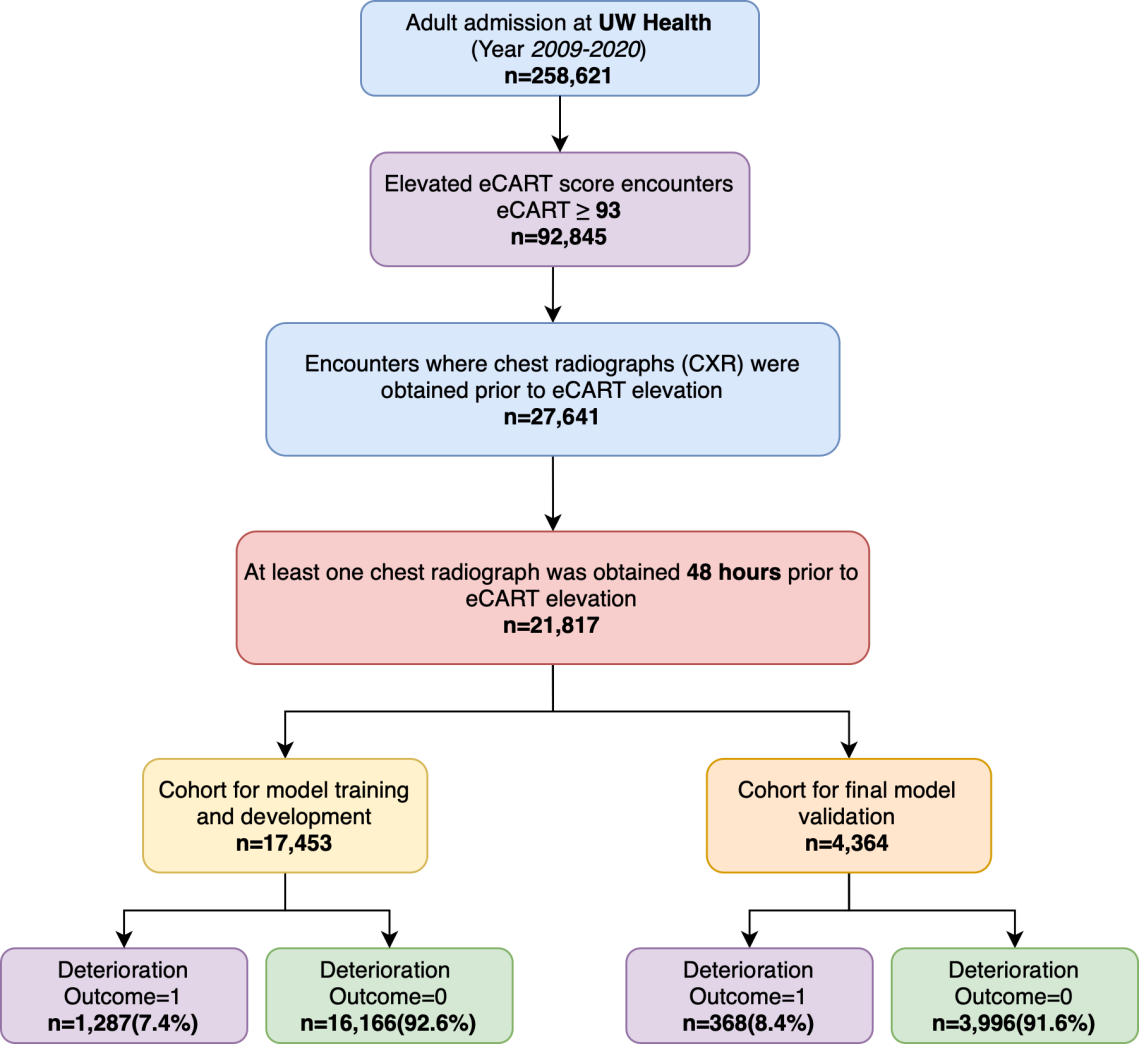
Study inclusion criteria flow diagram.

All adult patients (age ≥18 years) hospitalized at the University of Wisconsin Health System (UW Health) between 2009 and 2020 with an elevated eCART score ≥93 (which is the threshold used in clinical practice at UW Health) on the medical-surgical wards were eligible for inclusion in this retrospective cohort study. The eCART score [3] is a validated EWS currently in clinical practice and cleared by the Food and Drug Administration that combines demographics, vital signs, and laboratory results in a gradient-boosted machine model to predict future clinical deterioration. The rationale for only including patients with an elevated score is based on creating an enriched cohort where chest radiograph models can enhance the prediction and mitigate the false-positive alerts from these scores. Furthermore, this simplifies the prediction task to a single time point, making it more feasible to compare multiple models and augmentation strategies. Patients with a chest radiograph within 48 hours before the first elevated eCART score were included in the study. Available anterior-posterior or posterior-anterior views were included in the study cohort. In addition to chest radiographs, additional study variables that were collected included patient demographics, admission time, vital signs, laboratory values, patient location, and discharge disposition, which were all collected via the clinical research data warehouse. Figure 1 shows the patient encounter flow chart for inclusion into the analytic cohort.

### Outcome

The study outcome of clinical deterioration was defined as a direct ward-to-ICU transfer or ward death within 24 hours of the time of the patient’s first elevated eCART score.

### Data Preprocessing

The chest radiograph closest to (but before) the time of the elevated eCART score was used to predict the corresponding deterioration outcome. To address variations in image acquisition and processing protocols, all radiographs were rescaled to a uniform size of 224×224 pixels using nearest neighbor interpolation. Additionally, to address the variabilities in imaging exposure levels, pixel intensity values were normalized to a range of [0, 1] by applying min-max scaling. The clinical deterioration outcome (ie, ICU transfer or mortality within 24 hours from the prediction time point) was encoded as binary labels, with one-hot encoding used for the binary prediction task. These preprocessing steps ensured the creation of a high-quality robust dataset for training deep learning models to predict clinical deterioration from chest radiographs.

### Model Development

For the prediction task, computer vision deep learning models were trained and optimized with the dataset created from the cohort. Five publicly available computer vision models were compared for our task: (1) VGG16 [24], (2) DenseNet121 [25], (3) Vision Transformer [26], (4) ResNet50 [27], and (5) Inception V3 [28]. DenseNet121 is a convolutional neural network notable for its dense connections between layers, improving efficiency and reducing risk of overfitting, and VGG16 is known for its simplicity using a series of convolutional layers with small filters followed by max pooling layers. The Vision Transformer model is based on the transformer architecture and uses the self-attention mechanism to process the images. The main rationale of adopting these computer vision models for clinical deterioration tasks is that they are widely used in other chest radiograph detection tasks in clinical setups [29-31]. In addition, these models are easy to implement, and various pretrained weights are readily available. As clinical tasks require fine-grained image understanding for different tasks, these models provide that performance with a manageable model size. However, the main shortcoming of using these models is they do not provide any generalized image understanding for explainability.

**Figure 2.**
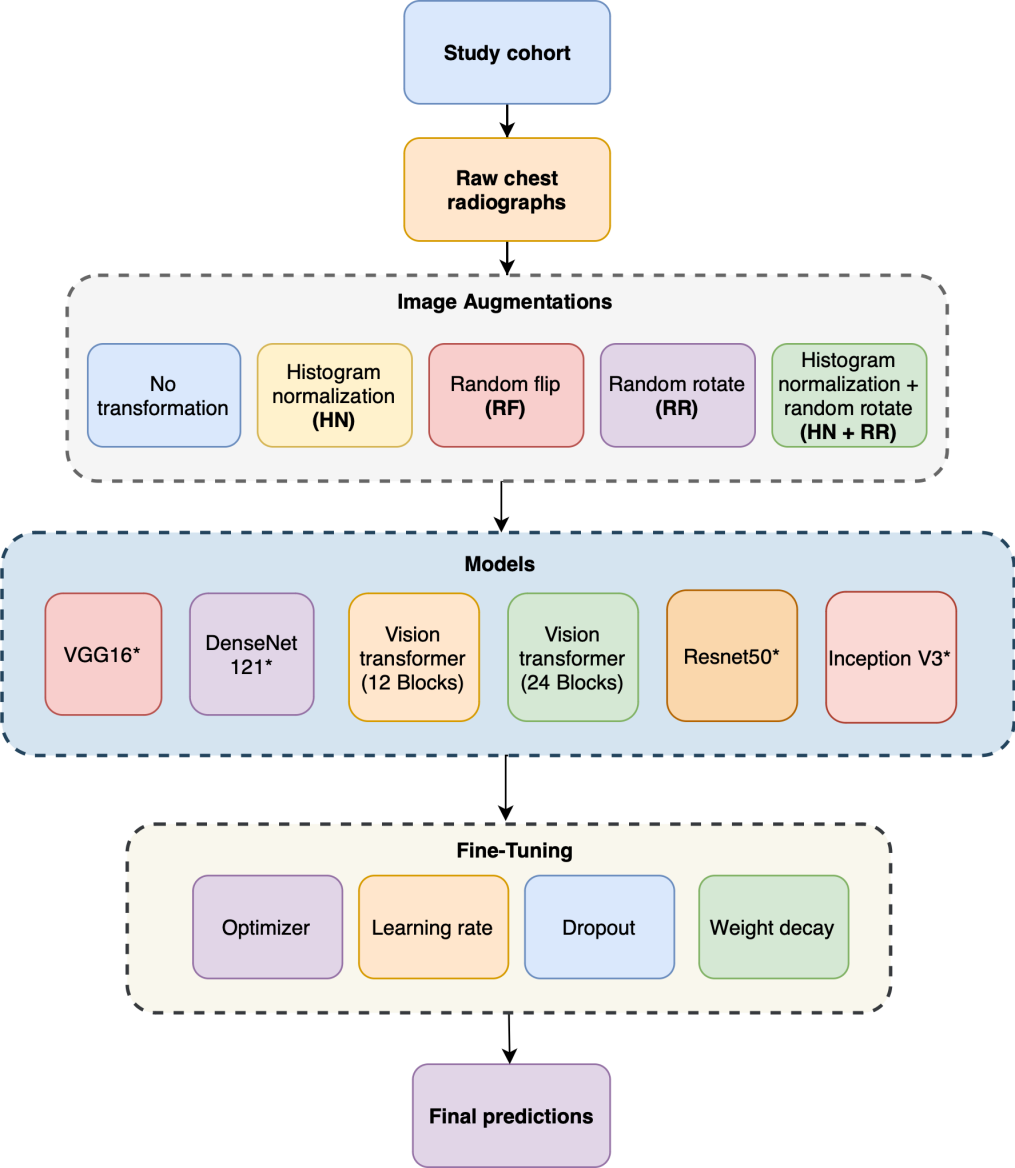
Overall structure of this study. *VGG16, DenseNet121, ResNet50, and Inception V3 models were trained from randomly initialized weights and pretrained weights. Other models were trained with randomly initialized weights only.

We used two different versions of the VGG16 architecture, one using randomly initialized weights (without pretraining) and the other using model weights pretrained on ImageNet [32]. Two different versions of the DenseNet121 architecture were also used: one with model weights pretrained on Imagenet [32] and one pretrained on publicly available radiograph datasets [33]. Specifically, the radiograph datasets used for pretraining consisted of the following datasets: NIH aka Chest X-ray14 [34], PC aka PadChest [35], CheX aka CheXpert [36], MIMIC-CXR [37], OpenI [38], Google [39], and RSNA Pneumonia Detection Challenge [40]. For the Vision Transformer model, we trained two models without any pretrained weights of two different sizes, one with 12 transformer blocks and another with 24 transformer blocks. We employed batch normalization layers after every block to ensure the stability of the optimization process during the model training. Figure 2 presents the overall structure of this study.

**Figure 3.**
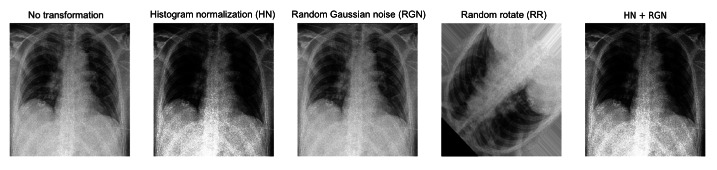
Examples of different image augmentation methods we have utilized. HN: histogram normalization; RGN: random Gaussian noise.

For each of the above architectures, we compared them with and without different preprocessing and data augmentation approaches. These included histogram normalization, random rotation (±15 degrees), horizontal flipping, and the addition of random Gaussian noise. Briefly, histogram normalization addresses the regional discrepancy of exposure levels in the case of some images. Additionally, given the presence of noise and artifacts during the acquisition of the radiographs, random Gaussian noise, which was implemented as 0.1 probability with 0 mean and 0.1 standard deviation, may make the models more robust to noise in the input image samples. Figure 3 shows the examples of all the augmentation methods we have used in this work.

We used the Bayesian optimization algorithm to find the optimal hyperparameters that maximize the area under the receiver operating characteristic curve (AUROC). Details of the hyperparameters are presented in Multimedia Appendix 1. To make the training procedure faster, we used Ray Tune [41] to parallelize the hyperparameter search process in a multi-GPU environment. We trained the model with a randomly selected 60% of the encounters in the dataset and validated it with the development set consisting of 20% of the encounters to optimize hyperparameters and determine the final settings. The remaining 20% of the encounters were completely separated for independent final model evaluation of the optimized models as a test set. We trained the models for 20 epochs and decreased the learning rate by a factor of 0.5 in every epoch. During the training, early stopping was used if the validation AUROC failed to improve in three consecutive epochs. We used Adam mini-batch gradient descent optimization with a batch size from the search space of 32, 64, and 128.

### Model Evaluation

All combinations of image augmentations and deep learning computer vision architectures for the clinical deterioration task were evaluated using the test dataset. Predicted probabilities for the deterioration outcome were calculated for every encounter during the evaluation. Model discrimination was assessed using the AUROC and its 95% CI, calculated via the DeLong method [42] as the primary metric and the area under the precision-recall curve (AUPRC) as the secondary metric. The p-values of the AUROC scores are presented in Multimedia Appendix 1. As *P*<.001 in all cases, our AUROC scores are statistically significant.

Data cleaning and cohort selection with descriptive analysis were conducted using Stata version 16.1 (StataCorp). We used Python version 3.8.10, along with the Monai framework version 1.2.0 (NVIDIA) and Pytorch version 2.0.0 (Facebook) to develop the deep learning models. Additionally, the AUROC score and its 95% CI were calculated using FastDeLong implementation from VMAF (Video Multimethod Assessment Fusion; Netflix) [43].

## Results

### Cohort Characteristics

A total of 258,621 admissions occurred during the study period, and 92,845 had an elevated eCART score. Of these, for 21,817 admissions, a chest radiograph was obtained within 48 hours of the time of the elevated score and was included in the analysis (Figure 1). The characteristics of the final cohort are presented in Table 1. The patients in the final cohort had a median age of 63 (IQR 52-74) years, with a higher likelihood of being male (56.1%, 12,249/21,817); 5.7% were black (1252/21,187). The median time to eCART score elevation from admission was 21.8 (7.1-47.6) hours and the median time to eCART score elevation from the last radiograph was 9 (7.1-47.6) hours. About 7.5% (1655/21,817) of the encounters had an outcome event, including 4.1% (893/21,817) cases of in-hospital death.

**Table 1.** Population characteristics of the study cohort (N=21,817).

Variable	Value
Age, years, median (IQR)	63 (52-74)
Female, n (%)	9568 (43.9)
Black race, n (%)	1252 (5.7)
Elevated eCART^a^ score, n (%)	1655 (7.59)
Time to the elevated eCART score from admission, hours, median (IQR)	21.8 (7.1-47.6)
Time to the elevated eCART score from the last radiograph, hours, median (IQR)	9.0 (7.1-47.6)
In-hospital mortality, n (%)	893 (4.1)

a eCART: electronic cardiac arrest risk triage

### Model Discrimination

**Figure 4.**
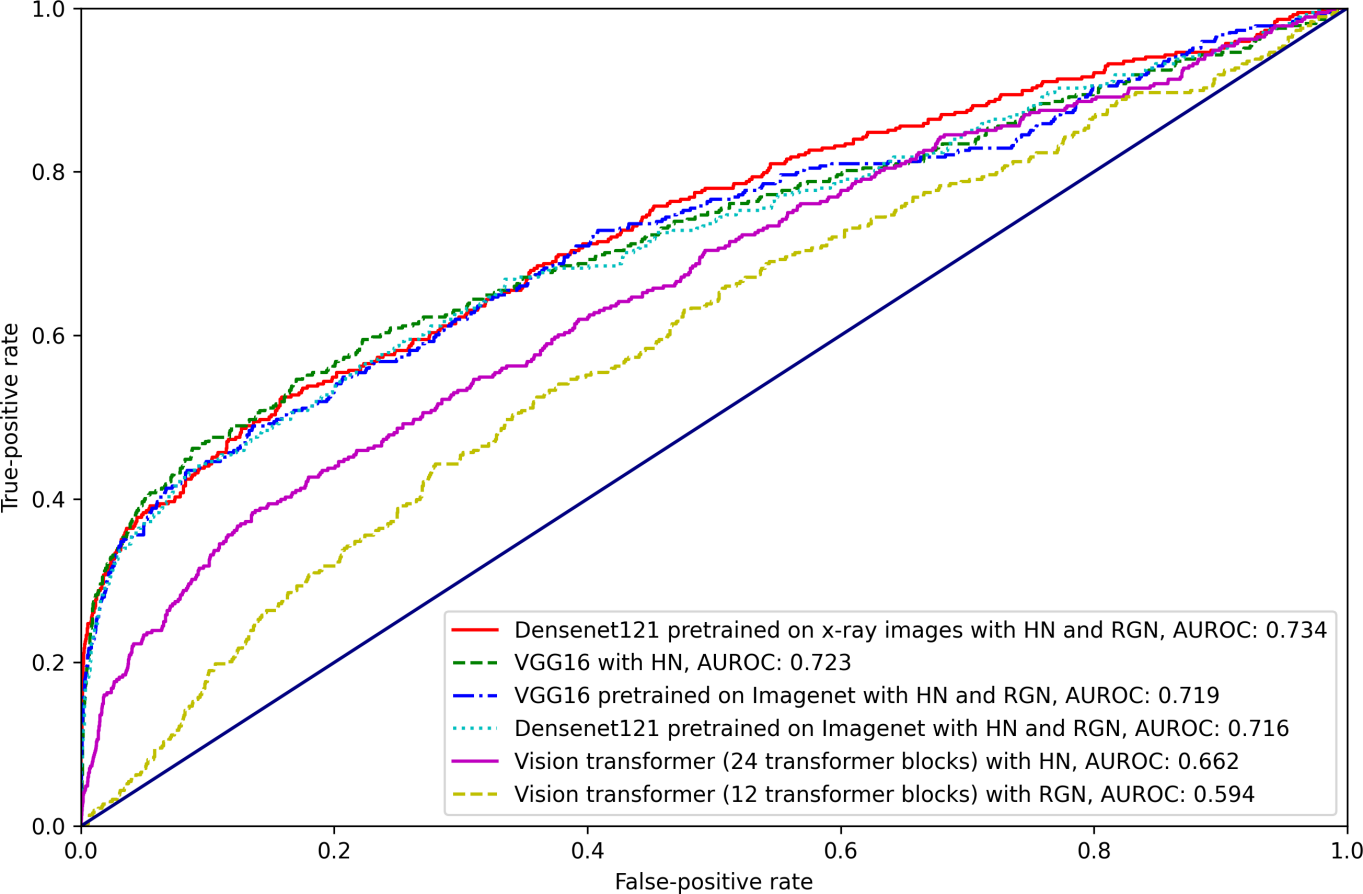
Receiver operating characteristic (ROC) curve of the best-performing models in every network architecture. Actual AUROC values are included in the corresponding label. HN: histogram normalization; RGN: random Gaussian noise; AUROC: area under the receiver operating characteristic curve.

The model performance AUROC and AUPRC values for all models across all image augmentation methods are presented in Tables2  3, respectively, and the 95% CI of the AUROC and AUPRC are presented in Multimedia Appendix 1. Additionally, receiver operating characteristic (ROC curves and precision-recall curves are shown in Figures4  5, respectively.

**Table 2.** Model performance area under the receiver operating characteristic curve (AUROC) with the validation dataset across different model architectures, pretrained weights, and image augmentation methods.

Model	Pretrained weights	No transformation	Histogram normalization (HN)	Random flip	Random Gaussian noise (RGN)	Random rotate	HN + RGN	Average AUROC score^a^
VGG16	Random init	0.694	0.723	0.698	0.701	0.674	0.712	0.700
VGG16	ImageNet	0.712	0.717	0.692	0.710	0.689	0.719	0.707
DenseNet121	ImageNet	0.683	0.701	0.672	0.700	0.678	0.716	0.692
DenseNet121	Radiographs	0.723	0.716	0.713	0.696	0.701	0.734	0.714
ResNet50	Random init	0.588	0.684	0.629	0.678	0.638	0.651	0.645
ResNet50	ImageNet	0.715	0.707	0.694	0.694	0.669	0.712	0.700
Inception V3	Random init	0.691	0.672	0.671	0.661	0.703	0.690	0.681
Inception V3	ImageNet	0.714	0.712	0.710	0.706	0.686	0.713	0.707
Vision Transformer (12 Blocks)	Random init	0.661	0.648	0.617	0.652	0.623	0.652	0.642
Vision Transformer (24 Blocks)	Random init	0.654	0.663	0.609	0.651	0.598	0.662	0.640
Average Score over models^b^	—^c^	0.684	0.694	0.671	0.685	0.666	0.696	—
Average Improvement^d^	—	—	0.010	−0.013	0.001	−0.028	0.012	—

a The average AUROC score is for a particular model over different augmentation methods

b The “Average score over models” row presents the average AUROC score of a particular augmentation method over different models.

c "—” indicates not applicable.

d The “Average improvement” row shows the average AUROC improvement of an augmentation method over the baseline score without any transformation.

**Table 3.** Model performance area under the precision-recall curve (AUPRC) scores with the validation dataset across different model architectures, pretrained weights, and image augmentation methods.

Model	Pretrained weights	No transformation	Histogram normalization (HN)	Random flip	Random Gaussian noise (RGN)	Random rotate	HN + RGN	Average AUPRC score^a^
VGG16	Random init	0.346	0.398	0.329	0.349	0.320	0.378	0.353
VGG16	ImageNet	0.371	0.403	0.306	0.343	0.311	0.389	0.354
DenseNet121	ImageNet	0.321	0.373	0.360	0.355	0.365	0.379	0.359
DenseNet121	Radiographs	0.395	0.326	0.338	0.360	0.358	0.414	0.365
ResNet50	Random init	0.135	0.229	0.147	0.243	0.215	0.174	0.191
ResNet50	ImageNet	0.405	0.378	0.357	0.320	0.288	0.344	0.349
Inception V3	Random init	0.319	0.247	0.247	0.304	0.343	0.339	0.300
Inception V3	ImageNet	0.440	0.340	0.421	0.399	0.361	0.369	0.388
Vision Transformer (12 Blocks)	Random init	0.205	0.189	0.143	0.209	0.139	0.204	0.182
Vision Transformer (24 Blocks)	Random init	0.187	0.219	0.121	0.177	0.118	0.196	0.170
Average score over models^b^	—^c^	0.313	0.310	0.277	0.306	0.282	0.319	—
Average improvement^d^	—	—	−0.003	−0.036	−0.007	−0.031	0.006	—

a The average AUPRC score is for a particular model over different augmentation methods

b The “Average score over models” row presents the average AUROC score of a particular augmentation method over different models.

c "—” indicates not applicable.

d The “Average improvement” row shows the average AUROC improvement of an augmentation method over the baseline score without any transformation.

**Figure 5.**
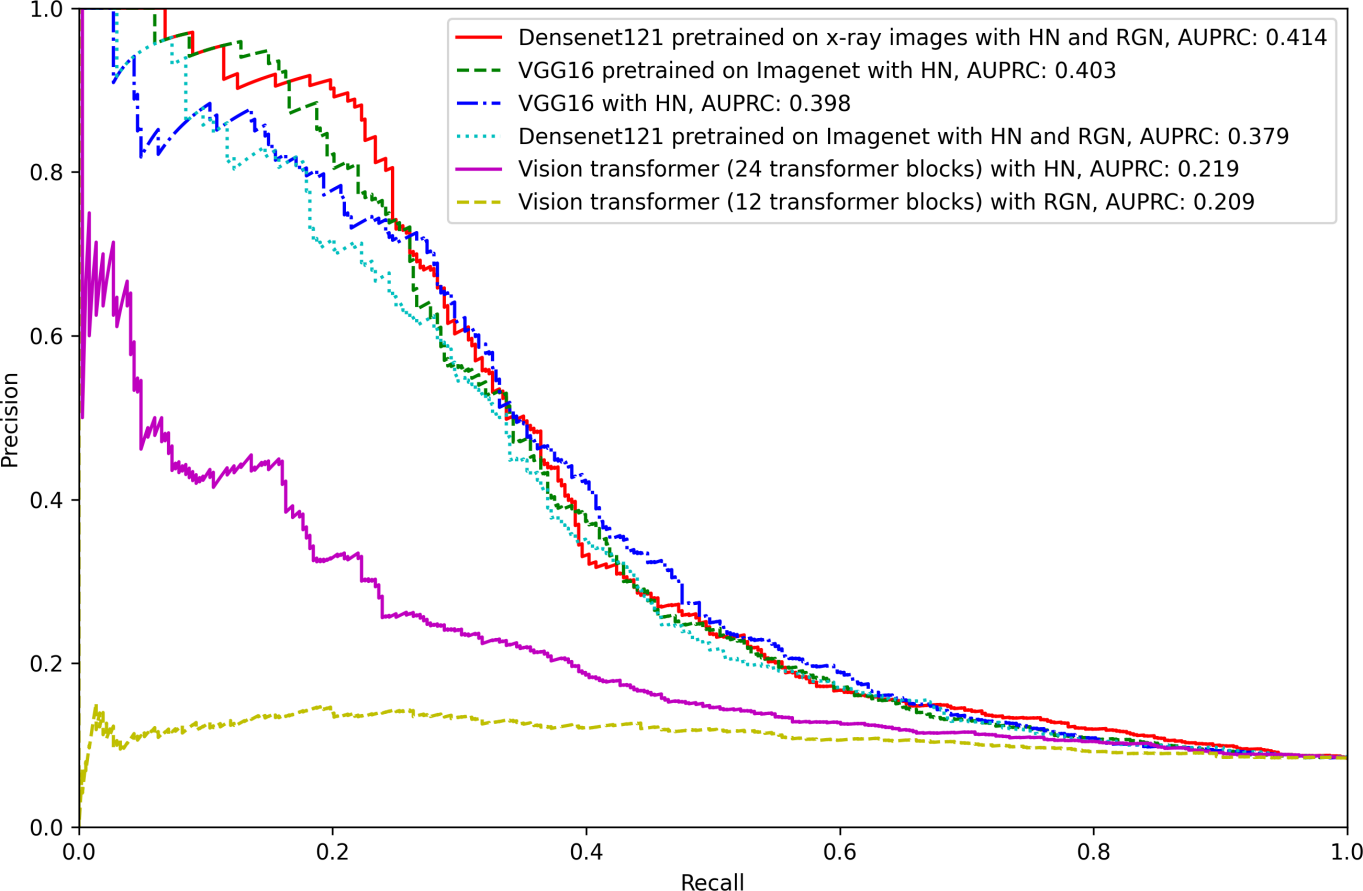
Precision-recall curves of the best-performing models in every network architecture. Actual AUPRC values are included in the corresponding label. Best viewed in color. HN: histogram normalization; RGN: random Gaussian noise; AUPRC: area under the precision-recall curve.

Across all architectures and augmentation combinations, the DenseNet121 model pretrained with chest radiographs and augmented with histogram normalization and Gaussian noise had the highest AUROC (0.734) across all the models. Similarly, when averaged across all augmentation methods, the DenseNet121 models pretrained with chest radiographs had a higher average discrimination than any other architecture in terms of the AUROC (0.714). The vision transformer architectures (12 and 24 transformer blocks) performed similarly to each other on average and had worse average AUROC than other models (0.642 and 0.640 for 12 and 24 transformer blocks, respectively). In terms of the AUPRC, DenseNet121 pretrained with chest radiographs and augmented with histogram normalization and Gaussian noise also had the highest performance (0.414). Accordingly, compared with other models, Inception V3 pretrained with ImageNet had the highest AUPRC (0.388) on average.

In terms of the image augmentation methods, the histogram normalization with random Gaussian noise image augmentations had the best mean AUROC (0.696) when averaged across all architectures, followed by histogram normalization augmentation alone (0.694). The random rotate augmentation had the worst average performance in terms of the AUROC (0.666). In terms of the AUPRC, histogram normalization with random Gaussian noise image augmentations also had the highest average AUPRC (0.319) across the models, and the models with no transformation alone had the next highest average AUPRC of 0.310. Unlike the AUROC results, the random flip augmentation had the worst AUPRC among all the four other augmentation methods.

## Discussion

### Principal Findings and Comparison With Previous Works

In this retrospective study with over 20,000 hospital admissions, we compared three deep learning computer vision architectures and four image augmentation methods for the early detection of clinical deterioration. We found that the DenseNet121 model pretrained on different publicly available chest radiographs had better discrimination than the VGG16 and Vision Transformer models based on the average AUROC metric. Among different image augmentation methods, a combination of histogram normalization and random Gaussian noise augmentations achieved higher AUROCs and AUPRCs on average than random flip and random rotate transformation. In all of the cases, we found that random flip and random rotate transformation lowered the discrimination compared to the baseline model in terms of both AUROC and AUPRC metrics. To the best of our knowledge, this is the first study to compare different computer vision models and image augmentation methods for predicting clinical deterioration outside the ICU. These findings have important implications in the field of using deep learning models to correctly identify patients showing clinical deterioration and to improve existing EWS applications in health systems.

Although DenseNet121 pretrained on chest radiographs achieved the maximum discrimination with histogram normalization and random Gaussian noise data augmentation, our investigation found multiple models exhibiting competitive performance across different data augmentation methods considering the AUROC. This may be due to our extensive hyperparameter search with Bayesian optimization that enables all models to achieve similar performances. Overall, the pretrained models performed better with respect to the models trained from scratch. This is consistent with the existing literature, as pretrained models already learned the fundamental building blocks of features (eg, lines and shades) from large number of images of the pretrained dataset [44,45]. However, as the VGG16 model was pretrained on the ImageNet [32] dataset, which is a collection of thousands of general-purpose images, and our dataset only contains chest radiographs, there may be a domain gap present in this scenario that prohibits the maximum benefits of the pretraining network. To analyze and mitigate that domain gap, we compared the performance of the DenseNet121 network pretrained on ImageNet and on a collection of the radiograph dataset. In almost all of the cases, DenseNet121 pretrained on radiographs outperformed the DenseNet121 model pretrained on ImageNet in terms of both the AUROC and AUPRC metrics. These experimental results proved our hypothesis and provided important insights into the use of pretrained networks with chest radiograph datasets. A prior study involving the classification of chest radiographs also found DenseNet networks achieving superior performance [10], which aligns with our findings. For example, Alhudhaif et al found that DenseNet201 achieved the highest discrimination in determining COVID-19 pneumonia with chest radiographs [10]. However, another work by Sitaula et al found that the VGG-16 model performed better than the DenseNet121 model for the classification of COVID-19 chest radiographs [11]. This discrepancy may be explained by differences in hyperparameter settings and the use of pretrained weight initialization. They tuned the hyperparameters manually, whereas we tuned the hyperparameters automatically with Bayesian optimization. As the DenseNet121 network is deeper than VGG-16 in terms of the number of layers, better hyperparameter tuning may enable DenseNet121 to learn more complex relationships without overfitting, hence achieving better performance than the VGG-16 network. Although DenseNet121 has more layers than VGG-16, DenseNet121 has fewer parameters than VGG-16 (7.98M vs 138.36M parameters). This parameter efficiency may reduce the risk of overfitting, which is important in medical imaging applications where datasets are often small. We also found that the Vision Transformer model underperformed in almost all the cases compared to other CNN-based models in the clinical deterioration prediction task. This finding contrasts with the recent success of Vision Transformer in general computer vision tasks [46]. However, in the case of classification tasks with radiographs, the lack of pretraining may harm the performance of the Vision Transformer models [47]. For the networks where we compared performance with random initialization and a pretrained model, in most of the cases, the pretrained model performed better than the randomly initialized one. This could be the main cause for the underperformance of the Vision Transformer models in our work, as we trained it from scratch.

In this study, we found that the models trained with histogram normalization combined with random Gaussian noise among different image augmentation methods achieved better performance, exhibiting the highest AUROC four times and the highest AUPRC three times for different architectures with different combinations of pretraining methods. However, the other two augmentation methods, random flip and random rotate, actually worsened the performance. Our findings align with the existing literature presenting performance improvements with histogram normalization and Gaussian random noise. Gielczyk et al showed that the combination of histogram normalization and Gaussian random noise achieved higher performances than the baseline method in detecting COVID-19 and pneumonia with chest radiographs [48]. However, this can be task dependent involving the useful features of that particular task. Lakhani et al presented a deep convolutional neural network for determining the presence and position of endotracheal tubes where random rotation and random flip augmentation achieved higher performances over the baseline values [12]. As that task was geometry-dependent, regularization introduced by random rotate and random flip augmentation might improve the performance. In contrast, our task of predicting clinical deterioration is not geometry dependent and hence did not benefit from geometric transformations like random rotate and random flips. These insights might be helpful in selecting appropriate image augmentation techniques in models involving chest radiographs.

### Strengths

Our study has several important strengths. First, our study cohort consisted of elevated-risk patients with an eCART score ≥93. Predicting deterioration in these patients is more challenging due to their rapid and unpredictable progressions compared to lower-risk patients. Second, we compared multiple deep learning architectures to evaluate their efficacy in predicting clinical deterioration. This comparative approach allows for a more robust understanding of a model’s performance in this context. Furthermore, by testing different data augmentation methods, the study explores ways to improve model performance. This aspect is crucial for enhancing the generalizability and robustness of the models. Incorporating Bayesian optimization with a large search space provides the models to achieve the most optimal performance.

### Limitations

Our study also has some limitations. First, we only considered the latest radiograph for our models to avoid bias and complexity. Although we reasoned that the latest radiograph conveys the most updated features of patients, prior radiographs and trends over time might carry important features for the model to predict clinical deterioration. Second, we focused on a few popular deep learning architectures with four different augmentation methods. Although recent studies have introduced numerous computer vision architectures, a more comprehensive study would be difficult considering our study’s dataset size. Third, in the deterioration prediction model, we only considered the features on chest radiographs. Incorporating other modalities, such as structured data and clinical notes, could improve the accuracy and robustness of our models and will be an interesting future work. Finally, even though our study is the largest of its kind, this was a single-center study, and future studies in other centers are needed to evaluate the external validity of our models.

### Conclusion

Our study demonstrates that the DenseNet121 model pretrained on chest radiographs often outperforms VGG16 and the Vision Transformer model with chest radiographs for the prediction of clinical deterioration. Furthermore, we found that model performance improves with histogram normalization along with random Gaussian noise augmentation in most models in terms of both the AUROC and AUPRC metrics. These results show that accurate prediction of patient clinical deterioration is feasible by utilizing chest radiographs while offering valuable insights into the use of computer vision-aided risk prediction.

## Supplementary material

10.2196/67144 Multimedia Appendix 1 Supplementary tables and figures.

## Abbreviations

AUPRC: area under the precision-recall curve
AUROC: area under the receiver operating characteristic curve
eCART: electronic cardiac arrest risk triage
EWS: early warning scores
ICU: intensive care unit
UW Health: University of Wisconsin Health System
VMAF: Video Multimethod Assessment Fusion
