# Supplementary material for: Comparison of Deep Learning Approaches Using Chest Radiographs for Predicting Clinical Deterioration: Retrospective Observational Study
Source: JMIR AI. 2025 Apr 10;4:e67144. doi: 10.2196/67144 (PMC12223691; doi:10.2196/67144)
Supplement: Multimedia Appendix 1 [file ai-v4-e67144-s001.docx]

**Using Chest Radiographs for Predicting Clinical Deterioration: Comparison of Deep Learning Approaches**

**SUPPLEMENTARY MATERIALS:**

**eSection 1: HYPERPARAMETER TUNING AND MODEL TRAINING PROCEDURE:**

In this section, we will provide a detailed explanations of the hyperparameter tuning and model training procedures used in this work. We used Ray Tune framework to optimize hyperparameters by training multiple models in parallel, each with a sampled hyperparameter set from a predefined search space. While training, the Ray Tune framework evaluates model performance based on the area under the receiver operating characteristic curve (AUROC) and dynamically manages the training process in real time with monitored metrics. Based on the evaluation, poorly performing models are automatically terminated early while ensuring that computational resources are focused on models with higher potential.

To further improve the efficiency of the training process and avoid unnecessary training, we implemented early stopping through the “ExperimentPlateauStopper” class of Ray Tune. This object monitors the top-performing models during training and halts further iterations if performance improvements plateau. Specifically, if the AUROC score did not improve by more than 0.001 standard deviations for 3 consecutive epochs, training for that model was stopped.

This approach prioritizes the exploration of effective hyperparameter configurations while reducing overfitting. Additionally, we capped the maximum number of epochs at 20, as the models either met the early stopping criteria or reached convergence well before the epoch limit. By combining parallelized training, real-time performance monitoring, and an effective early stopping mechanism, the Ray Tune framework proved a robust and computationally efficient method for identifying optimal hyperparameter settings.

**eTable 1: Interquartile Range (95% CI) for AUROC score for different models with different augmentation methods.**

| **Model (Pre-train)** | **No Transform** | **Histogram Norm (HN)** | **Random Flip (RF)** | **Random Gaussian Noise (RGN)** | **Random Rotate (RR)** | **HN + RGN** |
| --- | --- | --- | --- | --- | --- | --- |
| **VGG16** | 0.694  [0.661,  0.727] | 0.723 [0.691, 0.756] | 0.698 [0.666, 0.730] | 0.701 [0.668, 0.734] | 0.674 [0.640, 0.707] | 0.712 [0.679, 0.744] |
| **VGG16 (Imagenet)** | 0.712 [0.679, 0.745] | 0.717 [0.684, 0.749] | 0.692 [0.645, 0.712] | 0.710 [0.678, 0.742] | 0.689 [0.656, 0.721] | 0.719 [0.687, 0.751] |
| **DenseNet121 (Imagenet)** | 0.683 [0.651, 0.715] | 0.701 [0.668, 0.734] | 0.672 [0.639, 0.706] | 0.700 [0.668, 0.733] | 0.678 [0.645, 0.710] | 0.716 [0.684, 0.748] |
| **DenseNet121 (Radiograph Images)** | 0.723 [0.691, 0.754] | 0.716 [0.686, 0.747] | 0.713 [0.681, 0.745] | 0.696 [0.664, 0.728] | 0.701 [0.669, 0.733] | 0.734 [0.703, 0.765] |
| **Resnet50** | 0.588 [0.556, 0.620] | 0.684 [0.653, 0.716] | 0.629 [0.597, 0.660] | 0.678 [0.646, 0.710] | 0.638 [0.605, 0.671] | 0.651 [0.620, 0.682] |
| **Resnet50 (ImageNet)** | 0.715 [0.683, 0.748] | 0.707 [0.675, 0.739] | 0.694 [0.660, 0.727] | 0.694 [0.660, 0.727] | 0.669 [0.637, 0.702] | 0.712 [0.680, 0.743] |
| **Inception V3** | 0.691 [0.658, 0.723] | 0.672 [0.641, 0.703] | 0.671 [0.640, 0.703] | 0.661 [0.628, 0.694] | 0.703 [0.671, 0.736] | 0.690 [0.658, 0.722] |
| **Inception V3 (ImageNet)** | 0.714 [0.681, 0.748] | 0.712 [0.681, 0.744] | 0.710 [0.678, 0.742] | 0.706 [0.674, 0.738] | 0.686 [0.654, 0.719] | 0.713 [0.681, 0.745] |
| **Vision Transformer (12 Blocks)** | 0.661 [0.628, 0.693] | 0.648 [0.616, 0.680] | 0.617 [0.586, 0.648] | 0.652 [0.620, 0.683] | 0.623 [0.592, 0.656] | 0.652 [0.620, 0.684] |
| **Vision Transformer (24 Blocks)** | 0.654 [0.621, 0.686] | 0.663 [0.631, 0.694] | 0.609 [0.578, 0.639] | 0.651 [0.618, 0.683] | 0.598 [0.567, 0.629] | 0.662 [0.630, 0.694] |

**eTable 2: Interquartile Range (95% CI) for AUPRC score for different models with different augmentation methods.**

| **Model (Pre-train)** | **No Transform** | **Histogram Norm (HN)** | **Random Flip (RF)** | **Random Gaussian Noise (RGN)** | **Random Rotate (RR)** | **HN + RGN** |
| --- | --- | --- | --- | --- | --- | --- |
| **VGG16** | 0.346  [0.305, 0.387] | 0.398 [0.353, 0.442] | 0.329 [0.297, 0.378] | 0.349 [0.310, 0.390] | 0.320  [0.259, 0.340] | 0.378 [0.333, 0.422] |
| **VGG16 (Imagenet)** | 0.371 [0.324, 0.415] | 0.403 [0.360, 0.447] | 0.306 [0.267, 0.353] | 0.343 [0.298, 0.388] | 0.311 [0.256, 0.339] | 0.389 [0.345, 0.434] |
| **DenseNet121 (Imagenet)** | 0.321 [0.277, 0.360] | 0.373 [0.327, 0.416] | 0.360 [0.319, 0.404] | 0.355 [0.311, 0.394] | 0.365 [0.309, 0.399] | 0.379 [0.337, 0.423] |
| **DenseNet121 (Radiograph Images)** | 0.395 [0.354, 0.441] | 0.326 [0.281, 0.368] | 0.338 [0.321, 0.405] | 0.360  [0.299, 0.385] | 0.358 [0.309, 0.396] | 0.414 [0.368, 0.453] |
| **Vision Transformer (12 Blocks)** | 0.205 [0.173, 0.237] | 0.189 [0.161, 0.218] | 0.143 [0.132, 0.176] | 0.209 [0.175, 0.244] | 0.139 [0.128, 0.172] | 0.204 [0.153, 0.209] |
| **Vision Transformer (24 Blocks)** | 0.187 [0.159, 0.218] | 0.219 [0.183, 0.254] | 0.121 [0.107, 0.138] | 0.177 [0.152, 0.204] | 0.118 [0.101, 0.132] | 0.196 [0.167, 0.230] |

**eTable 3: Detailed implementation parameters of different data augmentation methods**

| **Augmentation Method** | **Attribute** | **Value** |
| --- | --- | --- |
| **Histogram Normalization** | Application Probability | 1 |
|  | Value range | [0, 256] |
|  | Number of histogram bins | 256 |
| **Random Flip** | Application Probability | 0.1 |
|  | Flip Axes | Randomly Selected from Horizontal or Vertical |
| **Random Gaussian Noise** | Application Probability | 0.1 |
|  | Mean value of Gaussian Noise | 0.0 |
|  | Standard Deviation of Gaussian Noise | 0.1 |
| **Random Rotate** | Application Probability | 0.2 |
|  | Rotation Range | [-15°, 15° ] |
|  | Interpolation mode | Bilinear |
|  | Padding mode | Border |

**eTable 4: List of hyperparameters and their respective search range in the developed training pipeline.**

| **Network Architectures** | **Hyperparameter** | **Type: Range** |
| --- | --- | --- |
| **VGG16** | Learning Rate | Float: [1e-4, 1e-2] |
|  | Weight decay | Float: [1e-5, 1e-1] |
|  | Batch Size | Choice: (32, 64, 128) |
|  | Dropout | Float: [0.2, 0.6] |
|  | Optimizer | Adam |
|  | Learning Rate Scheduler | StepLR, Step size=int: [1, 5], Gamma=0.5 |
|  | Early Stopper | Patience=int: [0, 6], Std=0.001 |
| **DenseNet121** | Spatial Dimensions | Int: 2 |
|  | In Channel | Int: 1 |
|  | Out Channels | Int: 2 |
|  | Learning Rate | Float: [1e-4, 1e-2] |
|  | Weight decay | Float: [1e-5, 1e-1] |
|  | Batch Size | Choice: (32, 64, 128) |
|  | Dropout | Float: [0.2, 0.6] |
|  | Optimizer | Adam |
|  | Learning Rate Scheduler | StepLR, Step size=[1, 5], Gamma=0.5 |
|  | Early Stopper | Patience= int: [0, 6], Std=0.001 |
| **Vision Transformer** | Number of heads | Choice: (12, 24) |
|  | In Channels | Int: 1 |
|  | Patch Size | Choice: (9, 16) |
|  | Spatial Dimensions | Int: 2 |
|  | Learning Rate | Float: [1e-4, 1e-2] |
|  | Weight decay | Float: [1e-5, 1e-1] |
|  | Batch Size | Choice: (32, 64, 128) |
|  | Dropout | Float: [0.2, 0.6] |
|  | Optimizer | Adam |
|  | Learning Rate Scheduler | StepLR, Step size=[1, 5], Gamma=0.5 |
|  | Early Stopper | Patience= int: [0, 6], Std=0.001 |
|  | Learning Rate | Float: [1e-4, 1e-2] |
|  | Weight decay | Float: [1e-5, 1e-1] |
|  | Batch Size | Choice: (32, 64, 128) |
|  | Dropout | Float: 0 |
|  | Optimizer | Adam |
|  | Learning Rate Scheduler | StepLR, Step size=[1, 5], Gamma=0.5 |
|  | Early Stopper | Patience= int: [0, 6], Std=0.001 |

**eTable 5: *P*-values for AUROC score for different models with different augmentation methods.**

| **Model (Pre-train)** | **No Transform** | **Histogram Norm (HN)** | **Random Flip (RF)** | **Random Gaussian Noise (RGN)** | **Random Rotate (RR)** | **HN + RGN** |
| --- | --- | --- | --- | --- | --- | --- |
| **VGG16** | <.001 | <.001 | <.001 | <.001 | <.001 | <.001 |
| **VGG16 (Imagenet)** | <.001 | <.001 | <.001 | <.001 | <.001 | <.001 |
| **DenseNet121 (Imagenet)** | <.001 | <.001 | <.001 | <.001 | <.001 | <.001 |
| **DenseNet121 (Radiograph Images)** | <.001 | <.001 | <.001 | <.001 | <.001 | <.001 |
| **Vision Transformer (12 Blocks)** | <.001 | <.001 | <.001 | <.001 | <.001 | <.001 |
| **Vision Transformer (24 Blocks)** | <.001 | <.001 | <.001 | <.001 | <.001 | <.001 |
| **BAEViT** | <.001 | <.001 | <.001 | <.001 | <.001 | <.001 |
